# Transiently expressed CRISPR/Cas9 induces wild-type dystrophin in vitro in DMD patient myoblasts carrying duplications

**DOI:** 10.1038/s41598-022-07671-w

**Published:** 2022-03-08

**Authors:** Veronica Pini, Virginie Mariot, Julie Dumonceaux, John Counsell, Helen C. O’Neill, Sarah Farmer, Francesco Conti, Francesco Muntoni

**Affiliations:** 1 Dubowitz Neuromuscular Centre, Molecular Neurosciences Section, Developmental Neuroscience Research and Teaching Department, UCL Great Ormond Street Institute of Child Health, London, WC1N 1EH UK; 2 Translational Myology Laboratory, Molecular Neurosciences Section, Developmental Neuroscience Research and Teaching Department, UCL Great Ormond Street Institute of Child Health, London, WC1N 1EH UK; 3 Genome Editing and Reproductive Genetics Group, Institute for Women’s Health, University College London, 86-96 Chenies Mews, London, WC1E 6HX UK; 4 NIHR Great Ormond Street Hospital Biomedical Research Centre, Great Ormond Street Institute of Child Health, University College London, & Great Ormond Street Hospital Trust, London, UK

**Keywords:** Genetic transduction, Transfection, Targeted gene repair, Cell delivery

## Abstract

Among the mutations arising in the *DMD* gene and causing Duchenne Muscular Dystrophy (DMD), 10–15% are multi-exon duplications. There are no current therapeutic approaches with the ability to excise large multi-exon duplications, leaving this patient cohort without mutation-specific treatment. Using CRISPR/Cas9 could provide a valid alternative to achieve targeted excision of genomic duplications of any size. Here we show that the expression of a single CRISPR/Cas9 nuclease targeting a genomic region within a *DMD* duplication can restore the production of wild-type dystrophin in vitro. We assessed the extent of dystrophin repair following both constitutive and transient nuclease expression by either transducing DMD patient-derived myoblasts with integrating lentiviral vectors or electroporating them with CRISPR/Cas9 expressing plasmids. Comparing genomic, transcript and protein data, we observed that both continuous and transient nuclease expression resulted in approximately 50% dystrophin protein restoration in treated myoblasts. Our data demonstrate that a high transient expression profile of Cas9 circumvents its requirement of continuous expression within the cell for targeting *DMD* duplications. This proof-of-concept study therefore helps progress towards a clinically relevant gene editing strategy for in vivo dystrophin restoration*,* by highlighting important considerations for optimizing future therapeutic approaches.

## Introduction

Duchenne Muscular Dystrophy (DMD) is a severe and incurable neuromuscular disorder affecting 1 in 5000 individuals worldwide^1^. DMD is caused by mutations arising in the *DMD* gene encoding dystrophin, a cytoskeletal protein essential for stabilizing the sarcolemma and connecting the inner muscle fiber cytoskeleton with the extracellular matrix^2^. When dystrophin is defective or absent, muscle fibres degenerate and patients become wheelchair dependent by their early teens. Cardiac and respiratory complications due to dystrophin absence in heart, diaphragm and intercostal muscles are responsible for the premature death of DMD patients, that generally occurs by the third decade of life^3,4^.

Intragenic deletions, duplications or point mutations are the main kind of mutations originating in *DMD* gene^5^. Within the large mutations, that are the most common in DMD patients (80%), 14% are multi-exon duplications^1^.

The mutation-agnostic therapeutic approaches under development, such as gene therapies using adeno-associated viral vectors (AAVs) as a vehicle to deliver a miniaturized but functional dystrophin molecule (mini/micro-dystrophin), could be applicable to all DMD patients including those carrying duplications. However, due to capacity of the AAVs, less than 50% of the coding sequence of the *DMD* gene can be delivered using this approach, and as such the resulting internally deleted dystrophin isoform cannot totally compensate for dystrophin absence. In the best scenario, this would result in a Becker-like phenotype, an allelic condition to DMD, with variable degrees of clinical severity^6^.

Exon skipping by antisense oligonucleotides, potentially applicable to 60–80% of patients with disrupted dystrophin reading frame secondary to deletions^7^, could theoretically also be used for duplications. Small duplications encompassing one or a small number of exons (such as *DMD* exon 2 and exons 2–7) were corrected in vitro in human myoblasts carrying *DMD* exon 2 duplication by using a phosphorodiamidate morpholino antisense oligonucleotide chemistry^8^. However, exon skipping proved to be challenging for the skipping of larger multi-exon duplications^9^. Moreover, when designing exon skipping antisense oligonucleotides for removing duplications, the level of skipping has to be carefully modulated, as excessive levels of skipping could eliminate both exon copies resulting in the transcription of a shorter mutated transcript, potentially also out-of-frame.

While both AAV-based gene therapies and exon skipping could potentially ameliorate DMD disease severity, none of them can address the *DMD* gene itself to permanently repair the mutation.

An alternative to these approaches could be found in engineered nucleases and in particular in CRISPR/Cas9, a bacterial-derived nuclease able to target and cleave any genomic region that needs editing by exploiting two specific components: single guide-RNA (sgRNA) and Cas9 nuclease^10^. Acting at the genomic level, any modification introduced by CRISPR/Cas9 in the target DNA is irreversible. Depending on the mutation, and specifically in patients with nonsense mutations or duplications, *DMD* editing via CRISPR/Cas9 would not result in a Becker-like dystrophin, but a fully functional wild-type dystrophin that would be expressed in a proportion of nuclei, resembling the situation of DMD carriers^11^. Depending on the extent of somatic cell correction, this could be advantageous as most DMD carriers are asymptomatic or have only mild clinical symptoms.

The Cas9 nuclease directed by a unique sgRNA to a duplicated *DMD* region would recognise and cleave two independent sites, resulting in the removal of the intervening duplicon and the restoration of the wild-type genomic sequence. This strategy has been explored before and single CRISPR/Cas9 constitutively expressed via integrating lentiviral vectors resulted in the permanent removal of the entire duplicon both in human fibroblasts (duplication of *DMD* exons 18–30) and myoblasts (*DMD* exon 2 duplication), restoring the mutated dystrophin to a wild-type state in vitro^12,13^ and thus showing the feasibility of such an approach.

In this study we aimed to compare a number of different strategies to induce correction of *DMD* duplications. Our results confirmed that a persistent nuclease expression in vitro is efficient in restoring the mutated dystrophin in patient-derived myoblasts carrying an in-frame *DMD* duplication and in addition demonstrated that a similar level of editing could be achieved even with a transient CRISPR/Cas9 expression.

## Results

### Restoration of in-frame dystrophin duplication in transduced patient-derived myoblasts expressing CRISPR/Cas9

**Figure 1:**
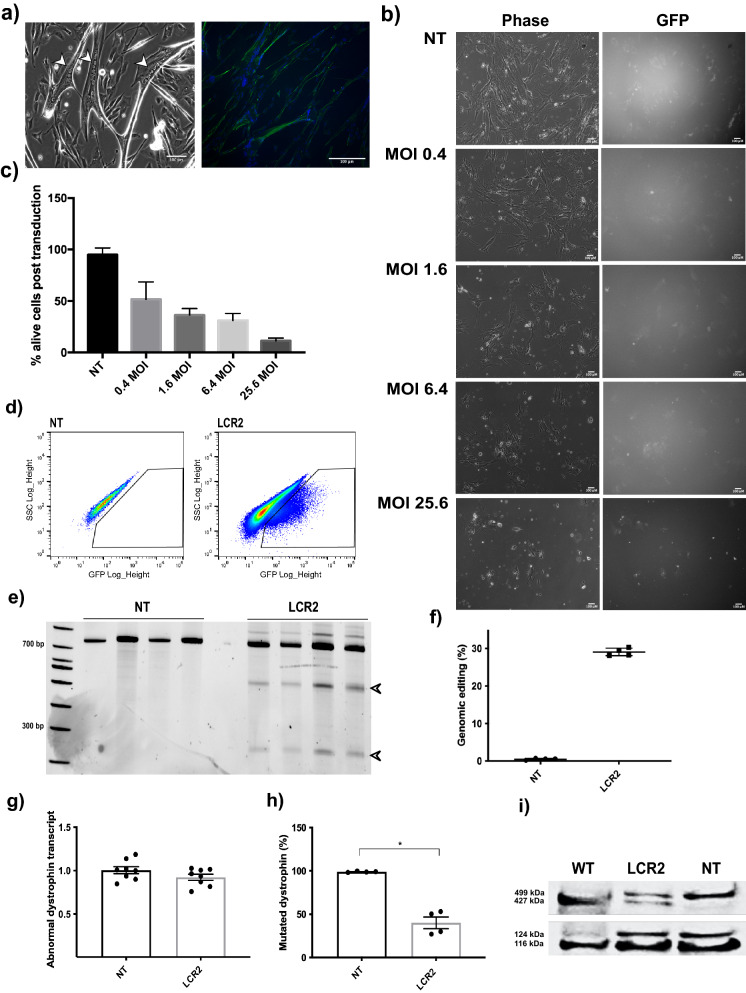
Restored wild-type dystrophin upon lentiviral transduction of CRISPR/Cas9 in DUP*myo* patient-derived myoblasts. (**a**) Multinucleation (arrows in left panel) and desmin expression (right panel) confirmed the high myogenicity and fusion index of *DUPmyo* cells (above 60%). (**b**) Fluorescent images of *DUPmyo* cells transduced with different MOIs of the lentiviral vector. Scale bar = 100 μm. (**c**) Percentages of viable cells identified following transduction with increasing MOIs. (**d**) FACS sorting of non-transduced (left panel) and LCR2-transduced (right panel) *DUPmyo* (MOI 0.4). (**e**) Replicates of T7E1 assay performed in non-transduced and transduced *DUPmyo* DNA amplicons (n = 4). Arrows indicate cleaved bands of expected size in amplicons targeted by the nuclease. (**f**) Percentage of *DUPmyo* cells/replicate in which genome cleavage was identified following LCR2 transduction with MOI 0.4. (**g**) Duplicated dystrophin transcript in NT and LCR2 (Mann–Whitney test, p = 0.28) (n = 2 technical replicates/sample). (**h**) Reduction of mutated dystrophin protein in LCR2 transduced *DUPmyo* cells (Mann–Whitney test, p-value = 0.0286) (n = 4)). (**i**) Western Blot showing both mutated (499 kDa) and wild-type dystrophin (427 kDa) in samples obtained from transduced *DUPmyo* cells. WT and NT samples represented positive and negative control, respectively, for monitoring the expression of wild-type dystrophin. Vinculin (116 KDa band) and meta-vinculin (124 kDa band) were probed as a loading control and a measure of myogenic differentiation, respectively. NT = untreated cells. WT = protein derived from the immortalized murine H2K 2B4 cells, expressing wild-type dystrophin, LCR2 = transduced cells.

We identified a highly myogenic primary myoblast cell line (DUP*myo*) (Fig. 1a) carrying an in-frame duplication of DMD exons 3–16, that results in the production of a mutated dystrophin protein of higher molecular weight (499 kDa) than wild-type dystrophin (427 kDa). In this cell line, the mutated, higher molecular weight protein provides a means to benchmark gene editing efficacy, by normalising full-length dystrophin expression to the duplicated protein by semi-quantitative densitometric analysis of Western blots.

We designed nucleases targeting intronic regions within the *DMD* gene duplicon, considering that the prevalent repair mechanism of DNA breaks (non-homologous end-joining)^14^ could randomly introduce insertions and/or deletions (indels) at the break site and potentially disrupt the *DMD* gene reading frame if occurring in an exon^15^. We screened sgRNAs targeting four regions of *DMD* intron 9 (Supplementary Table 1a), which lies within the exon 2–20 hotspot where about 5% of genomic breakpoints are detected in DMD patients carrying duplications^16^. The targeted genomic regions were chosen based on the rarity of polymorphisms that could lead to a reduction or loss in the efficiency of the nucleases^17^. sgRNA2 showed the best cleavage efficiency in HEK293T cells (35.3% ± 0.56), followed by sgRNA1 (31.8% ± 0.2) and sgRNA4 (29.8% ± 0.41), while sgRNA3 failed to generate cleaved products of the expected size (Supplementary Fig. 1).

We hypothesised that permanent, constitutive expression of CRISPR/Cas9 components from integrated lentiviral vector genomes should increase the rate of cleaving dystrophin DNA at both duplicated sites, consequently restoring as much dystrophin protein as possible. We cloned sgRNA2 under the U6 promoter into an integrating lentiviral vector (LCR2) that co-expresses Cas9 and enhanced GFP (EGFP), facilitating the quantification and purification of positively transduced cells. We generated lentiviral particles expressing LCR2 and transduced *DUPmyo* with increasing viral doses ranging from a multiplicity of infection (MOI) 0.4 to 25.6 (Fig. 1b). Non-transduced cells were used as a negative control for the transduction, and a positive control to monitor cell viability. EGFP-positive cells were observed with the highest MOI, although this viral load was toxic as only 11.3% ± 2.6 cells were alive following transduction. On the other hand, we found that the lowest viral dose (MOI 0.4), despite inducing only a very faint fluorescence signal, resulted in the best cell viability (51.7% ± 16.8 live cells) (Fig. 1c). We therefore selected the MOI dose of 0.4, as this dose allowed nuclease expression with a minimal toxicity for myoblasts compared to other tested dosages. We considered that this degree of transduction could result in a more controlled level of nuclease expression across cells, as each transduced cell should be limited to a single integrated lentiviral vector genome per cell. This low viral yield would also be safer, limiting the risks associated with insertional mutagenesis^18^. EGFP-positive cells transduced at 0.4 MOI were isolated via fluorescence activated cell sorting (FACS) (mean transduction efficiency of 4.48% ± 0.14) (Fig. 1d). Most of the gated fluorescent cells were close to non-targeted cells, consistent with EGFP being expressed at low levels. Cleaved bands of the expected molecular size of 511 bp and 217 bp were seen in transduced samples but not in untreated cells (Fig. 1e). The observed genomic cleavage, which had an efficiency of 29.0% ± 0.45 (Fig. 1f) resulted in an efficient protein editing in transduced cells, despite the mutated dystrophin transcript showing a limited trend towards reduction. In fact, the wild-type dystrophin in cells expressing the nuclease accounted for approximately 50% of the total dystrophin protein (59.9% ± 6.74) (Fig. 1g–i and Supplementary Fig. 2). This result confirmed that integrating lentiviral particles can successfully deliver and express CRISPR/Cas9 in primary patient-derived myoblasts, remove multi-exon duplications within the *DMD* gene and ensure a high level of dystrophin correction in these cells.

### Nuclear electroporation is the most efficient non-viral method to deliver CRISPR/Cas9 to patient-derived myoblasts

**Figure 2:**
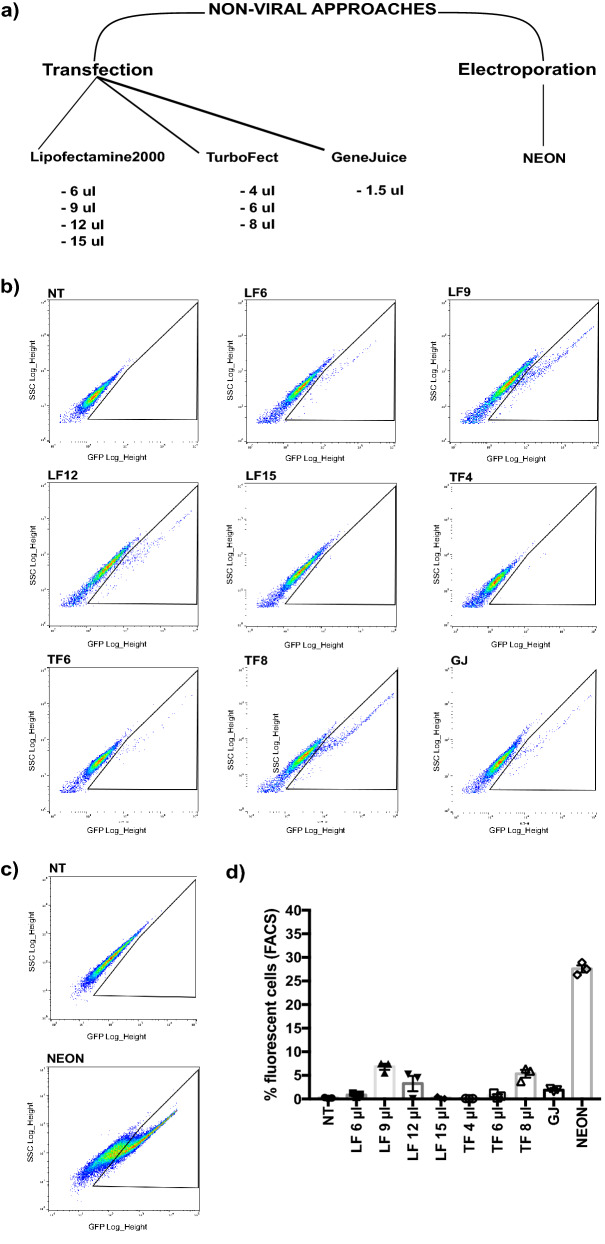
Screening of non-viral methods to deliver CRISPR/Cas9 in primary myoblasts. (**a**) Schematic of tested non-viral technologies and used doses. (**b**) FACS analysis of pCMV-GFP plasmid expression in *DUPmyo* cells following transfection. Lipofectamine2000 (LF), TurboFect (TF) and GeneJuice (GJ). Lipofectamine2000 doses were 6 μl (LF6), 9 μl (LF9), 12 μl (LF12) and 15 μl (LF15). TurboFect was tested at a dosage of 4 μl (TF4), 6 μl (TF6) and 8 μl (TF8). GeneJuice (GJ) was tested at a single concentration. (**c**) FACS analysis of *DUPmyo* cells electroporated with the pCMV-GFP plasmid using the NEON device. (**d**) Comparison of the efficiencies of tested techniques (n = 3/technique) for plasmid delivery in *DUPmyo* cells.

Given that integrating lentiviral vectors could produce undesirable long-term effects on treated cells such as random genomic integration^19^, we proceeded to investigate whether a transient CRISPR/Cas9 expression could be sufficient to mediate dystrophin editing. As part of this process, we tested and compared several non-viral methods for delivering CRISPR/Cas9 in vitro into patient-derived myoblasts (Fig. 2a).

Transfection efficacy in *DUPmyo* cells was first assessed using a GFP-expressing plasmid of 4735 Kb to facilitate the screening process. We assessed the efficiency of three commercial transfection reagents (Lipofectamine2000, TurboFect and GeneJuice) and measured their transfection efficiency via FACS analysis (Fig. 2b). These methods resulted in a variable, but generally poor, transfection efficiency (around 5% or lower). The highest transfection efficiency was observed when transfecting *DUPmyo* with 9 μl Lipofectamine 2000 (mean transfection efficiency 6.84% ± 0.64), confirming the work of Jackson e*t al.* in murine myoblasts^20^. 3.27% ± 1.62 fluorescent cells were detected when transfection was done with 12 μl Lipofectamine 2000, while doses of 6 μl and 15 μl Lipofectamine 2000 resulted in very low transfection rates (0.86% ± 0.26 and 0.18% ± 0.15, respectively). Transfection efficiency with TurboFect was comparable to that observed with 9 μl Lipofectamine 2000 when using 8 μl of reagent (5.3% ± 0.83). Only 0.083% ± 0.02 and 0.71% ± 0.29 fluorescent cells were seen upon *DUPmyo* transfection with 4 μl and 6 μl TurboFect. A poor transfection was also observed for GeneJuice, whose efficiency of transfection was less than 2% (1.89% ± 0.18). Taken together, these results confirmed the well-known difficulty of achieving a high level of transfection in primary myoblasts using common transfection reagent techniques^21,22^.

We next tested nuclear electroporation of the DNA plasmid using the Neon transfection system, which allows customization of electroporation parameters, such as the number of electric pulses, their duration and the voltage^23^. Almost 30% of cells (27.57 ± 0.75) were fluorescent following electroporation (Fig. 2c). As this percentage of targeted cells is at least five times higher than that achieved with the previously used transfection reagents (Fig. 2d), we decided to use NEON as a non-viral method to test the ability of an episomal Cas9 expression to correct dystrophin duplications in primary myoblasts.

### Transient CRISPR/Cas9 expression in immortalized myoblasts can correct dystrophin duplications with comparable efficacy to stable expression

**Figure 3:**
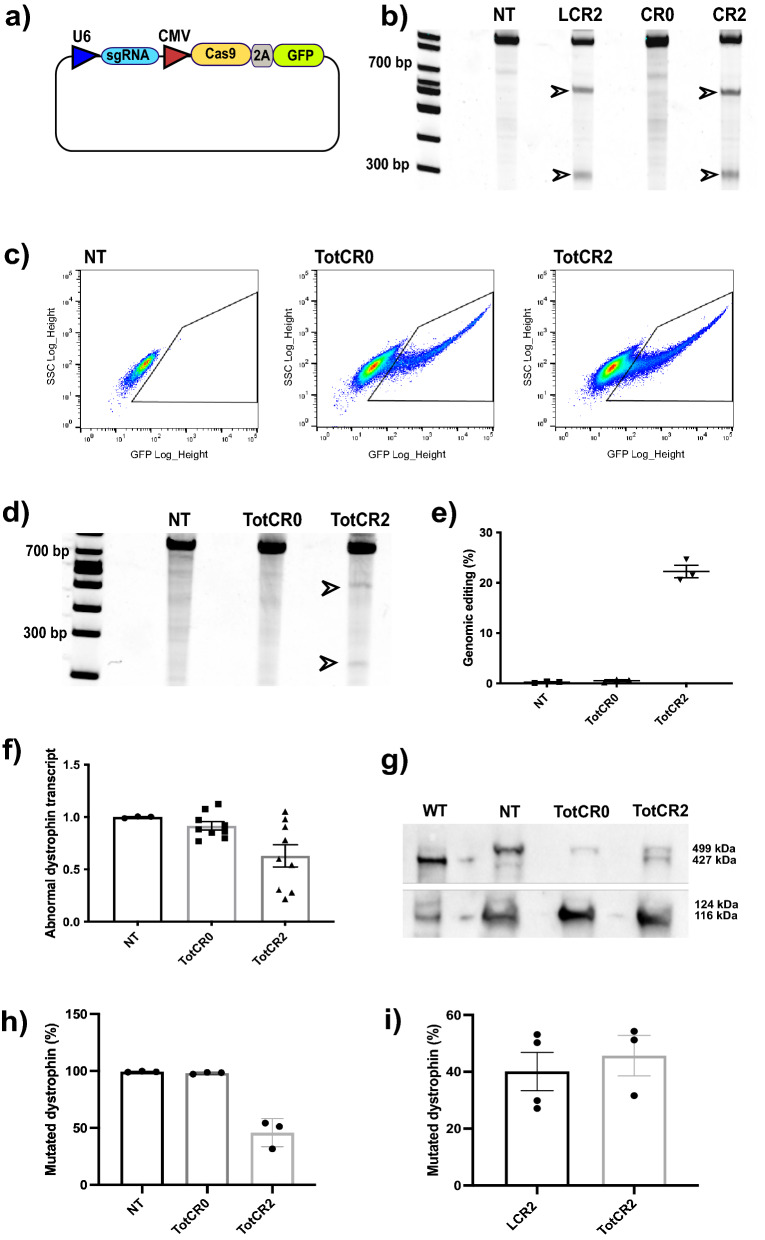
Dystrophin correction in immortalized DUPmyo cells electroporated with CRISPR/Cas9-expressing plasmids. (**a**) Schematic of the plasmid where CR0 and CR2 were cloned. (**b**) T7E1 assay performed on HEK293T transfected with the LCR2 plasmid (lentiviral vector expressing sgRNA2), CR0 (negative control plasmid) and CR2 (plasmid expressing sgRNA2). Arrows indicated cleaved bands of expected molecular size in cells expressing the nuclease. LCR2 was used as a reference to evaluate the targeting efficiency of CR2. CR0 did not show cleaved bands as LCR2 and CR2, proving it is a valid negative control for monitoring the targeting effect of CRISPR/Cas9. NT = untreated cells. (**c**) FACS analysis of immortalized *DUPmyo-i* myoblasts electroporated by NEON. (**d**) T7E1 assay performed on the total pool of electroporated *DUPmyo-i* cells expressing CR0 and CR2. (**e**) Efficiency of genomic targeting evaluated in T7E1 replicates (n = 3). (**f**) Mutated dystrophin transcript in cells expressing CR0 and CR2 (Kruskall-Wallis test, p = 0.0552) (n = 3 technical replicates/sample). (**g**) Western blot showing dystrophin correction (427 kDa band) in cells expressing CR2. Vinculin (116 KDa band) and meta-vinculin (124 kDa band) were probed as a loading control and a measure of myogenic differentiation, respectively. (**h**) Percentages of mutated dystrophin assessed in electroporated *DUPmyo-i* (Kruskall-Wallis test, p = 0.0679) (n = 3). i) Comparison of mutated dystrophin observed in patient-myoblasts following LCR2-transduction (n = 4) and CR2 electroporation (n = 3) (Mann–Whitney test, p = 0.4). NT = untreated cells. TotCR0/TotCR2 = total pool of cells expressing CR0/CR2. WT = protein derived from the immortalized murine H2K 2B4 cells, expressing wild-type dystrophin (positive control), LCR2 = transduced cells.

We next proceeded to test the efficiency of dystrophin gene editing by NEON electroporation. We delivered a DNA plasmid of 8279 Kb containing a sgRNA cassette, Cas9 and GFP (Fig. 3a). We used the universal negative control ‘CR0’ plasmid (targeting a sequence not present in the human genome) and CR2, which expresses sgRNA2. We first tested these plasmids in HEK293T cells and observed that the cleavage efficiency of electroporated CR2 (35.2%) was comparable to that of lentivirally-delivered LCR2 (29.9%) (Fig. 3b and Supplementary Fig. 3). We electroporated *DUPmyo* with CR0 and CR2 and isolated the CRISPR/Cas9 expressing cells by FACS. However, the excessive passaging needed to expand the sorted cells led them to become senescent before being induced towards terminal differentiation. Further optimization was therefore carried out in immortalized *DUPmyo* cells (*DUPmyo-i*)^24^. A lower transfection efficiency of *DUPmyo-i* was observed compared to primary cells (mean electroporation efficiencies of 13.27% ± 1.6% and 11.5% ± 0.6 for CR2 and CR0, respectively) (Fig. 3c). The levels of genomic targeting in sorted cells upon transient CRISPR/Cas9 expression (mean value of 22.2% ± 1.23) (Fig. 3d,e and Supplementary Fig. 4) were similar to those achieved in transduced *DUPmyo* expressing the nuclease (29.0% ± 0.45), with mutated dystrophin transcripts showing a trend towards reduction in cells expressing the nuclease (p = 0.0552) (Fig. 3f).

Most importantly, by densitometric analysis of dystrophin Western blot we observed that transient CRISPR/Cas9 expression by electroporation restored dystrophin to a wild-type state, (Fig. 3g and Supplementary Fig. 5) allowing a mean reduction of mutated dystrophin protein of 45.7% ± 6.10 (Fig. 3h). The amount of wild-type dystrophin detected following electroporation (54% ± 7.11) was comparable to that achieved in transduced cells (59.9% ± 6.74), suggesting that transient expression of CRISPR/Cas9 is an effective method to target duplicated DNA regions within dystrophin and efficiently restore the production of a wild-type protein (Fig. 3i).

### Contribution of Cas9 expression intensity to dystrophin editing

**Figure 4:**
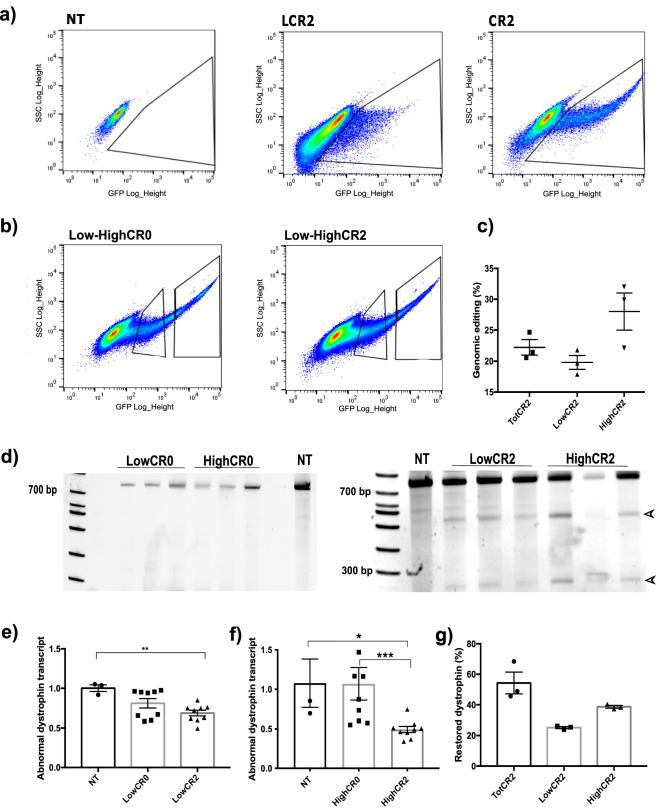
The intensity of transient Cas9 expression affects dystrophin restoration. (**a**) Comparison of CRISPR/Cas9 expression intensity by FACS analysis of transduced (LCR2) and electroporated *DUPmyo* (CR2). (**b**) FACS analysis of electroporated *DUPmyo-i* cells gated according to their GFP expression intensity (high/low). (**c**) Percentages of genomic targeting achieved in cell populations expressing different CRISPR/Cas9 levels (n = 3). (**d**) T7E1 assay of cells expressing high and low level of CR0 and CR2 nucleases (n = 3). Arrows indicate expected cleaved bands in cells expressing CR2. (**e**) Reduction of mutated dystrophin transcript observed in cells expressing both low (Kruskal–Wallis test: p = 0.028, Mann–Whitney test: p = 0.0091) and high (**f**) levels of CR2 (Kruskal–Wallis test: p = 0.0005, Mann–Whitney tests: p = 0.0182 and p = 0.0008 (n = 3 technical replicates/sample). (**g**) Comparison of average percentages of restored dystrophin observed in cells expressing TotCR, LowCR2 and HighCR2 (n = 3). NT = untreated cells. LowCR0/LowCR2 = cells expressing a low level of CR0/CR2. HighCR0/CR2 = cells expressing high levels of CR0/CR2. TotCR2 = total pool of cells expressing CR2. WT = protein derived from the immortalized murine H2K 2B4 cells, expressing wild-type dystrophin (positive control).

We noticed that, despite their similar efficiency of dystrophin restoration, transduction and electroporation differed in their frequency of GFP expression. Our data show that NEON electroporation resulted in at least three times more fluorescent cells than lentivirally-treated cells, although the majority of transduced cells (LCR2) tend to cluster close to the fluorescent-negative cell population, whereas FACS data obtained from electroporated *DUPmyo-i* (CR2) highlight the presence of a pool of cells expressing high levels of GFP (Fig. 4a). Hypothesizing that GFP expression correlates with CRISPR/Cas9 expression levels, we investigated whether GFP expression levels correlated with dystrophin editing efficacy.

We performed *DUPmyo-i* electroporation using CR0 and CR2 plasmids and by FACS analysis we gated GFP-positive cells according to their fluorescence intensity, which is representative of Cas9 expression levels. We named the two groups ‘Low’ and ‘High’ to indicate their degree of GFP expression and sorted them accordingly via FACS (Fig. 4b). The mean percentages of fluorescent cells in the Low populations were 8.37 ± 0.49 (CR0 Low) and 10.41 ± 0.8 (CR2 Low) whereas, in the High population, fluorescent cells accounted for 3.92 ± 0.28 (CR0 High) and 3.60 ± 0.39 (CR2 High).

As expected, we noted a dose response, with the percentage of genomic targeting reduced in the Low (19.8% ± 1.12) compared to the High population (28.0% ± 3.01) (Fig. 4c,d). We also observed a similar trend in the transcript, with the mutated transcript being more reduced in cells of the High population (Fig. 4e,f).

By averaging the transcript correction level of the Low and High cell populations expressing CR2 (0.58 ± 0.03) we obtained a similar mean level of transcript correction to that detected in the total pool of GFP-positive cells (0.62 ± 0.10), supporting the hypothesis that the level of editing seen in the total pool of cells expressing Cas9 is likely given by the contribution of cells expressing both high and low Cas9 levels. Wild-type dystrophin was also detected in both Low and High populations, although its amount was higher (38.6% ± 0.88) in cells expressing most Cas9 than in cells that poorly expressed Cas9 (25.23% ± 0.65) (Fig. 4g). This indicates that the intensity of a transient nuclease expression is a crucial factor to control for, as weak and strong Cas9 expression can achieve different levels of protein restoration.

## Discussion

Among the genetic mutations reported in the *DMD* gene, 10–15% are multi-exon duplications (1). A recently described strategy for the correction of *DMD* duplications in vitro relies on a single CRISPR/Cas9 nuclease designed to cleave a specific site within the *DMD* duplicon and expressed by integrating lentiviral vectors^12,13^. We confirmed the validity of this approach and compared the editing level of *DMD* duplications following either a permanent or transient nuclease expression in vitro, represented by myoblast transduction with integrating lentiviral vectors and electroporation, respectively.

Lentivirus was used as an exemplar for viral vector mediated CRISPR/Cas9 delivery, which is more commonly being explored as a therapeutic gene editing platform in the context of AAVs, as AAV vectors also showed long-term expression in muscle in vivo^25^. Electroporation, which is also being investigated for *DMD* gene editing^26,27^, proved to be the best among the transient transfection techniques we screened for delivering CRISPR/Cas9-expressing plasmids to primary myoblasts, and was therefore chosen to perform our proof-of-concept studies.

We first questioned whether non-viral CRISPR/Cas9 expression would be able to induce the repair of dystrophin duplications in vitro. We identified the Neon transfection system as an optimal method for in vitro delivery of CRISPR/Cas9 transgenes. Surprisingly but encouragingly, transient but efficient expression of CRISPR/Cas9 using the Neon system resulted in a similar efficiency of dystrophin correction to the stable, durable viral delivery system. Considering that in humans between 29 and 57% of wild-type dystrophin could completely prevent muscular dystrophy when expressed in the majority of muscle fibres within the muscle^28^, the level of dystrophin correction we achieved with both approaches (around 50%) could be sufficient to provide a therapeutic benefit, if obtained in vivo.

By analysing the amount of wild-type dystrophin protein in cells expressing either low or high levels of GFP, we observed that the cells which were mostly contributing to the mutated dystrophin repair were those with the highest fluorescence, that we hypothesize to have the highest nuclease levels. This observation provided the proof-of-concept that efficient, but transient, expression of Cas9 can be as effective as a weaker, constitutive expression. Therefore, as long as CRISPR/Cas9 is expressed at sufficient levels, it does not need to be constantly present within cells for efficient correction of *DMD* duplications.

From a therapeutic perspective, a strategy using viral vectors such as AAVs could have an advantage compared to a single transfection obtained with non-viral methods, as the persistent presence of the CRISPR/Cas9 nuclease in the cytoplasm could help the repair of new nuclei originating from the diseased muscles’ regenerative process. However, the increased number of muscle nuclei is inevitably coupled with focal muscle fibre degeneration and regeneration resulting from the high degree of muscle damage. This would likely lead to a dilution effect of the transgene, especially if administered in early childhood. As such, the ideal therapeutic formulation for DMD would be particularly efficacious if developed to also target satellite cells. Acting as muscle-specific stem cells, satellite cells expressing wild-type dystrophin would allow the replacement of damaged fibres with fully functional ones^29^. However, routinely used AAVs have a generally low tropism for satellite cells^30^. Also, the repeated administration of AAVs is currently precluded by the strong immune response observed upon repeated AAV injections^31^.

Delivering the nuclease via a non-viral method, instead, could circumvent such issue and, if required, be repeated. A minimal and temporary nuclease expression might also increase the tolerance of the immune system against Cas9 protein and limit the triggering of the immune response to the Cas9 itself, observed in healthy human adults^32^. Finally, reducing the half-life of Cas9 in the cytoplasm proved to be efficient in lowering the chances of observing off-target effects^33^.

The delivery of CRISPR/Cas9 via electroporation (which we tested in vitro as a proof-of-concept to represent non-viral techniques) could only be applied for ex vivo cell-based therapies. However, many other non-viral strategies such as lipid and protein-based nanoparticles are being considered for the delivery of the nuclease not only in vitro but also in vivo^34–36^. Recently developed nanoparticle formulations overcome the viral-mediated entry in the cells by relying on endocytosis, and proved to have a very limited toxicity, efficient genomic editing and high targeting specificity when administered intramuscularly in *mdx* mice^33^. These encouraging results suggest that such technologies could also become a valid choice also for a systemic CRISPR/Cas9 delivery, which would be the optimal option to treat any disorder such as DMD, that not only affects muscles body wise but also has a multisystemic involvement^37,38^.

Any in vivo CRISPR/Cas9-based therapeutic design should therefore follow a “hit-and-run” strategy for expression of Cas9^39,40^. Further optimizations to increase both the efficiency and safety of the single CRISPR/Cas9 nuclease approach, including an in-depth study of genomic off-target effects, will be needed before clinically translating CRISPR/Cas9 for DMD. However, it is highly likely that refinement of CRISPR/Cas9 technologies will proceed in conjunction with the rapidly evolving nonviral delivery systems, and the new knowledge obtained by the recently developed nanoparticles-based mRNA vaccines designed to fight the COVID-19 pandemic will hopefully accelerate the identification of safe and efficacious CRISPR/Cas9-based therapeutic formulations.

## Materials and methods

### Ethics statement

The work carried out in this project involved the manipulation of human cells obtained from the MRC Centre for Neuromuscular Diseases BioBank. Primary myoblasts used for this work were obtained from a DMD patient carrying an in-frame duplication of *DMD* exons 3–16. Clinically, this patient performs within the better end of the DMD spectrum. The disease manifested when the patient was 3 years old with global developmental delay; later on he was diagnosed with autistic spectrum disorder and aggressive behaviour. Since the age of 5, the patient has been taking steroids. He is currently 13 and still ambulant. The patient has a positive Gowers’ sign (+ 4 s), with only partial antigravity power in hip and shoulder proximal muscles.

Ethical approval and consent for research have been obtained to facilitate pharmacological, gene and cell therapy trials in neuromuscular disorders (NRES Committee London- West London & GTAC, REC reference number 06/Q0406/33) and to allow the use of cells as a model system to study pathogenesis and therapeutic strategies for neuromuscular disorders (NRES Committee London—Stanmore, REC reference 13/LO/1826), in compliance with national guidelines regarding the use of human-derived cell lines for research. Written informed consent was obtained from the patient’s guardians.

### sgRNA design and cloning in integrating lentiviral CRISPR/Cas9 expression vectors

The full intron 9 sequence of the human *DMD* gene was retrieved through the Ensemble genome browser (www.ensembl.org/). We selected genomic regions spanning from 176 to 225 bp throughout *DMD* intron 9, and run them through the algorithm developed by Feng Zhang laboratory at MIT (http://crispr.mit.edu/). For each genomic target, we chose the returned sgRNAs with the highest quality score (inversely correlated to the number of their potential off-target effects). Details about each genomic sequence and its genomic location are provided in Supplementary Table 1 together with the sequence of sgRNAs 1–4. sgRNAs were cloned into the integrating LentiCRISPRv1 plasmid (Addgene #49535) from Feng Zhang’s laboratory. The best sgRNA (sgRNA2) was also cloned into the integrating pL-CRISPR.EFS.GFP plasmid from Benjamin Ebert’s laboratory (Addgene #57818), following the specified protocol.

### Lipofectamine transfection of HEK 293T cells

HEK293T cells were plated in Dulbecco’s Modified Eagle’s Medium (DMEM) (Gibco) supplemented with 10% foetal bovine serum (FBS) (Life Technology). Plasmid transfection occurred using Lipofectamine 2000 (LF2000), at a ratio of 1 μg DNA to 2 μl LF2000. After 24 h, transfection medium was replaced with fresh culture medium and cells were kept in culture at 37 °C and 5% CO2 for further 24 h.

### Culture and differentiation of patient-derived myoblasts

Experiments were performed in muscle-derived primary *DUPmyo* myoblasts and *DUPmyo-i* cell line. *DUPmyo-i* is the result of *DUPmyo* myoblast immortalization, carried out by Dr. Vincent Mouly^24^. Primary and immortalized myoblasts were cultured in complete Skeletal Muscle Growth medium (PromoCell) containing 3 mM GlutaMax (Invitrogen), 10% (v/v) FBS and 40 μg/ml Gentamicin (Sigma). Cells were maintained at 37 °C and 5% CO2. Terminal differentiation of human myoblasts was achieved by culturing cells for 5–7 days with a confluence of 70% with DMEM (MegaCell), 2% (v/v) FBS, 1X non-essential amino-acids, 2 mM Glutamine, 0.5 mM, β-mercaptoethanol and 5 ng/ml basic fibroblast growth factor (bFGF).

### Desmin immunostaining and myogenicity assay

Terminally differentiated cells were fixed with 4% paraformaldehyde and permeabilized with Triton-X. After two phosphate-buffered saline (PBS) washes, 250 μl 10% goat serum was added and incubated for 30 min at room temperature. Desmin primary antibody (Dako, mouse anti-human) was diluted 1:100 in 10% goat serum and PBS. This was added to the cells and incubated for 60 min at room temperature. DAPI (Thermo Fisher Scientific) was also added (1:10,000) and incubated together with desmin antibody to stain the nuclei in each cell. Following three PBS washes, cells were incubated with the secondary antibody (Invitrogen, goat anti-mouse conjugated with Alexa488) prepared in PBS (1:100 dilution). Myogenicity was assessed by microscope analysis by measuring the ratio between the total number of desmin-positive cells with more than 3 nuclei, and the total number of DAPI stained nuclei.

### Production of lentiviral particles expressing functional CRISPR/Cas9

Lentiviral particles expressing pL-CRISPR.EFS.GFP plasmid were generated by transfecting HEK293T cells by means of FuGENE6 transfection reagent (Promega), using a DNA:FuGENE6 ratio of 1:3. Transfected plasmids included pL-CRISPR.EFS.GFP (16 μg/plate), psPax2 plasmid (Addgene #12260, 12 μg/plate) and pCMV-VSVg plasmid (Addgene #8454, 4 μg/plate). Transfection complexes were prepared according to the manufacturer’s instructions and added dropwise to the HEK293T cells. Transfected cells were incubated at 37 °C and 5% CO_2_ to allow the assembly and release of lentiviral particles into the culture medium. At days 3 or 4 post-transfection, cell culture medium was collected and filtered through a 40 μm filter (Corning) to remove cells and debris. Filtered media, containing lentiviral particles, was placed in 25 × 83 mm polyallomer centrifuge tubes (Beckman Coulter) and centrifuged for 2 h at 60,000×*g* at 4 °C in a Sorvall Discovery 90SE centrifuge. Following ultracentrifugation, viral particles were resuspended in Opti-MEM (Thermo Fisher Scientific), aliquoted and stored at -80 °C.

### Primary myoblasts transduction

Lentiviral particles (MOIs 0.4, 1.6, 6.4, 25.6) were added to the cells culture medium, rocking the plate to ensure homogeneous distribution. Cells were then incubated overnight at 37 °C and 5% CO2. The following day, cells were washed using PBS and cultured for 48 h in complete Skeletal Muscle Growth medium (PromoCell) containing 3 mM GlutaMax (Invitrogen), 10% (v/v) FBS (Life Technology) and 40 μg/ml Gentamicin (Sigma).

### Primary myoblasts transfection

Transfection of 2 × 10^5^ *DUPmyo* cells/well of a 6-well plate (10 × 10^3^ cells/cm^2^) was performed in serum-free Opti-MEM (Thermo Fisher Scientific) as indicated in manufactures’ instructions. pCMV-GFP plasmid (Addgene #11153) from Connie Cepko’s laboratory was used to test transfection methods. 2.5 μg of plasmid DNA was transfected in combination with different amounts of Lipofectamine 2000 (ThermoFisher Scientific) (6 μl, 9 μl, 12 μl and 15 μl). TurboFect transfection (ThermoFisher Scientific) was done with 2 μg of plasmid DNA mixed with different volumes of transfection reagent (4 μl, 6 μl and 8 μl). 2 μg DNA were also used to transfect cells with 6 μl of GeneJuice reagent (Sigma-Aldrich). After transfection, cells were incubated at 37 °C and 5% CO2 for the amount of time specified by each protocol. Finally, the transfection medium was removed and replaced by the complete Skeletal Muscle Growth medium (PromoCell) and transgene expression evaluated after 48 h.

### Nuclear electroporation of human myoblasts—NEON

5 × 10^5^ *DUPmyo* (or *DUPmyo-i*) myoblasts were electroporated with 1 μg of the highly pure CRISPR/Cas9-GFP plasmids by using the NEON device (Thermo Fisher Scientific) provided by Julie Dumonceaux and Virginie Mariot, as specified by the manufacturer instructions. Number of pulses, duration and voltage intensity were suggested by Dumonceaux and Mariot, as follows: 1 pulse, 20 ms, 1400 V. Electroporated plasmids were U6gRNA-CMVCas9-GFP (Sigma-Aldrich) expressing sgRNA0 and sgRNA2 (named CR0 and CR2).

### GFP sorting

Transduced/transfected/electroporated cells were washed with PBS and detached from the plate with trypsin–EDTA (Gibco). Cells were centrifuged at 500×*g* for 5 min at room temperature and washed with PBS. The pellet was resuspended in PBS and transferred into the appropriate Falcon round-bottom polystyrene FACS tubes (Thermo Fisher Scientific). Samples were then placed on ice and brought to the FACS facility (https://www.ucl.ac.uk/child-health/core-scientific-facilities-centres/flow-cytometry- core-facility), where trained personnel performed the FACS by either FACSAria III, FACSCalibur or MoFlow XDP Cell sorter. Sorted cells were then incubated overnight in complete Skeletal Muscle Growth medium (PromoCell), at 37 °C and 5% CO_2_.

### Evaluation of DNA editing

Genomic DNA was extracted by human cell lines using the DNeasy Blood & Tissue Kit (QIAgen). The genomic region surrounding the CRISPR/Cas9 target sites was amplified by touchdown PCR (tdPCR) performed with the high fidelity Q5 DNA polymerase (New England BioLabs). TdPCR parameters were 2 min at 98 °C, followed by 26 amplification cycles of 10 s at 98 °C, 30 s at 68 °C (− 0.5 °C per cycle) and 30 s at 72 °C each. Other 9 amplification cycles, each of 10 s at 98 °C, 30 s at 56 °C and 30 s at 72 °C were then added to complete the protocol.

DNA fragments were purified with the QIAquick PCR purification Kit (QIAgen). T7E1 endonuclease (New England BioLabs) assay was performed on 200 ng of purified tdPCR product according to the manufacturer’s instructions. T7E1 reaction stopped by the addition of 2 μl of 0.25 M EDTA was run on a Novex TBE gel (Life Technologies) as specified by the manufacturer. The TBE gel was then stained for 15 min with SybrGold (Life Technologies) diluted 10000X in 200 ml TBE, at room temperature on an orbital shaker in the dark. The gel was visualized with the Gel Doc XR + system (Bio-Rad). The efficiency of CRISPR/Cas9 genomic cleavage efficiency was quantified by exporting in Excel format the data from the densitometric band analysis, which was done by using Fiji software (https://imagej.net/Fiji). CRISPR/Cas9 cleavage efficiency, expressed in percentage, was calculated as follows: upper cleaved band area value/ (Upper cleaved band + Full-length band area values) × 100.

### Quantification of duplicated and wild-type dystrophin transcript

RNA was extracted from myotubes using the RNeasy Mini kit (QIAgen) and retrotranscribed to cDNA with the High-Capacity RNA-to-cDNA Kit (Thermo Fisher Scientific). qPCR was run by a StepOne device (Thermo Fisher Scientific) on 10 ng of cDNA as follows: 95 °C for 3 min, and 40 cycles of 10 s at 95 °C and 1 min at 60 °C. qPCR data were exported in Excel format and analysed using the ∆∆Ct method. To avoid the amplification of multiple dystrophin transcript isoforms, *DMD* exon 20 was chosen as anchor region to normalize and quantify dystrophin transcript correction, as it is located outside the duplication and before any of the dystrophin promoters throughout *DMD* gene. The following primers were used:

- Dystrophin_Exons8-9_Forward = 5’-TTGCCAAGGCCACCTAAA-3’,
- Dystrophin_Exons8-9_Reverse = 5’-TCTCTCATATCCCTGTGCTAGA-3’.
- Dystrophin_Exon20_Forward = 5’-TGGATCGAATTCTGCCAGTT-3’.
- Dystrophin_Exon20_Reverse = 5’GCTCCAATTGTTGTAGCTGATTAT-3’.

### Protein quantification and electrophoresis

Proteins were extracted from myotubes by replacing culture medium with NHC lysis buffer (4 M urea, 125 mM Tris pH 6.8 and 4% SDS) supplemented with 1X Complete Protease Inhibitor Cocktail Tablets (Roche). Samples were and left on ice for 10 min after which they were collected, boiled for 3 min and centrifuged at 14000×*g* for 10 min at 4 °C. Protein quantification was done with the Pierce BCA protein assay (Thermo Fisher Scientific). 50 μg of protein samples were run on a precast NuPAGE Novex Tris- Acetate 3–8% gradient gel (Invitrogen) according to manufacturer’s instructions. 10 μl of the HiMark pre-stained protein standard (Life Technology) and the Odyssey One-Color protein molecular weight marker (Licor) were included as a reference molecular weight to analyse the protein of interest. The gel was run on ice at 75 V for 45 min to allow dystrophin to slowly enter the gel and then at 150 V for 2 h and 15 min.

### Assessment of dystrophin protein restoration

At completion of the run, the gel was placed in contact with a polyvinylidene difluoride (PVDF) low-fluorescence membrane. Protein transfer was performed at 30 V for 3 h with the electrophoresis apparatus XCell Sure Lock Mini-Cell (Thermo Fisher Scientific). 300 ml of diluted Transfer buffer (270 ml distilled water, 15 ml 20X NuPAGE Transfer buffer and 15 ml methanol) were placed in the inner chamber, while the outer chamber was filled with 600 ml of cold water, both replaced after 1.5 h. The transfer apparatus was placed in ice to avoid overheating. Following blotting, the membrane was blocked with 10% non-fat milk powder (, OXOID) diluted in Tris buffered saline (TBS) for 1 h at room temperature. The PVDF membrane was then incubated overnight at 4 °C with 10 ml 5% non-fat milk diluted in TBS-Tween (TBS-T), which contained the primary rabbit anti-human dystrophin (Abcam, 1:200) and mouse anti-human vinculin (Sigma, 1:100.000) antibodies. Vinculin antibody protein was used to control if the samples to be analysed are differentiated to a similar extent. This antibody recognizes both vinculin and its slightly higher molecular weight isoform meta-vinculin, whose expression increases on skeletal muscle differentiation^41^. The membrane was then washed three times with TBS-T and incubated with biotinylated secondary antibodies (Abcam) (1:1000) for 1 h at room temperature. Following another three TBS-T washes, the membrane was incubated 1 h at room temperature with streptavidin-HRP (1:5000) (Thermo Fisher Scientific) in order to amplify the signal. The membrane was washed with TBS-T and incubated for 1 min with 2 ml of the Luminata Forte Western HRP substrate (Millipore). Signal was detected placing the membrane in a ChemiDoc Imager and densitometric analysis of detected bands was performed by using the ImageLab software (Bio-Rad). The amount of restored protein was calculated as the ratio between the amount of duplicated and wild-type dystrophin protein.

### Microscopy and image capture

The Olympus IX Inverted microscope (Olympus Life Science) and Leica DMR microscope (Leica Microsystem) were used to acquire images. Any image processing was done by Fiji software (https://fiji.sc/).

### Statistical analysis

Replicate experiments were expressed as the mean ± the standard error of the mean. Statistical analysis aimed to verify the effect of CRISPR/Cas9 treatment versus untreated controls were performed by means of the GraphPad Prism software. Results were analysed by applying the Mann–Whitney test (comparison between two groups) or Kruskal–Wallis (comparison between three groups or more) as, due the small sample size, data were assumed to be not normally distributed. Statistical significance was set at P-values below 0.05.

### Primer design

Primers were designed by using the *primer3* on-line tool (primer3.ut.ee/) and synthesized by Sigma-Aldrich. All primers were provided as desalted stocks which were diluted in pure water to a concentration of 100 μM and stored at -20 °C. When needed, they were further diluted in sterile water to a concentration of 10 μM. The list of all primers designed to amplify CRISPR/Cas9 targets is provided in Supplementary Table 2.

## Supplementary Information

Supplementary Information.

## Data Availability

All data generated or analysed during this study are included in this published article (and its Supplementary Information files).

## References

[1] Bladen CL (2015). The TREAT-NMD DMD global database: Analysis of more than 7,000 Duchenne muscular dystrophy mutations. Hum. Mutat..

[2] Hoffman EP, Brown RH, Kunkel LM (1987). Dystrophin: The protein product of the Duchenne muscular dystrophy locus. Cell.

[3] Fayssoil A, Abasse S, Silverston K (2017). Cardiac involvement classification and therapeutic management in patients with duchenne muscular dystrophy. J. Neuromuscul. Dis..

[4] Simonds AK, Muntoni F, Heather S, Fielding S (1998). Impact of nasal ventilation on survival in hypercapnic Duchenne muscular dystrophy. Thorax.

[5] Ferlini A, Neri M, Gualandi F (2013). The medical genetics of dystrophinopathies: Molecular genetic diagnosis and its impact on clinical practice. Neuromuscul. Disord..

[6] Monaco AP, Bertelson CJ, Liechti-Gallati S, Moser H, Kunkel LM (1988). An explanation for the phenotypic differences between patients bearing partial deletions of the DMD locus. Genomics.

[7] Rodino-Klapac LR, Mendell JR, Sahenk Z (2013). Update on the treatment of Duchenne muscular dystrophy. Curr. Neurol. Neurosci. Rep..

[8] Greer KL, Lochmüller H, Flanigan K, Fletcher S, Wilton SD (2014). Targeted exon skipping to correct exon duplications in the dystrophin gene. Mol. Ther. Nucleic Acids.

[9] Aartsma-Rus A, Janson AAM, van Ommen G-JB, van Deutekom JCT (2007). Antisense-induced exon skipping for duplications in Duchenne muscular dystrophy. BMC Med. Genet..

[10] Jinek M (2012). A programmable dual-RNA-guided DNA endonuclease in adaptive bacterial immunity. Science.

[11] Soltanzadeh P (2010). Clinical and genetic characterization of manifesting carriers of DMD mutations. Neuromuscul. Disord..

[12] Wojtal D (2016). Spell checking nature: Versatility of CRISPR/Cas9 for developing treatments for inherited disorders. Am. J. Hum. Genet..

[13] Lattanzi A (2017). Correction of the Exon 2 duplication in DMD myoblasts by a single CRISPR/Cas9 system. Mol. Ther. Nucleic Acids.

[14] Williams GJ (2014). Structural insights into NHEJ: Building up an integrated picture of the dynamic DSB repair super complex, one component and interaction at a time. DNA Repair. (Amst)..

[15] Lin M (2017). Effects of short indels on protein structure and function in human genomes. Sci. Rep..

[16] Juan-Mateu J (2015). DMD mutations in 576 dystrophinopathy families: A step forward in genotype-phenotype correlations. PLoS ONE.

[17] Hsu PD (2013). DNA targeting specificity of RNA-guided Cas9 nucleases. Nat. Biotechnol..

[18] Charrier S (2011). Quantification of lentiviral vector copy numbers in individual hematopoietic colony-forming cells shows vector dose-dependent effects on the frequency and level of transduction. Gene Ther..

[19] Howe SJ (2008). Insertional mutagenesis combined with acquired somatic mutations causes leukemogenesis following gene therapy of SCID-X1 patients. J. Clin. Invest..

[20] Jackson MF (2012). Genetic manipulation of myoblasts and a novel primary myosatellite cell culture system: Comparing and optimizing approaches. FEBS J..

[21] Quenneville SP (2004). Nucleofection of muscle-derived stem cells and myoblasts with ϕC31 integrase: Stable expression of a full-length-dystrophin fusion gene by human myoblasts. Mol. Ther..

[22] Ye L (2007). Nonviral vector-based gene transfection of primary human skeletal myoblasts. Exp. Biol. Med..

[23] Brees C, Fransen M (2014). A cost-effective approach to microporate mammalian cells with the Neon Transfection System. Anal. Biochem..

[24] Mamchaoui K (2011). Immortalized pathological human myoblasts: Towards a universal tool for the study of neuromuscular disorders. Skelet. Muscle.

[25] Buchlis G (2012). Factor IX expression in skeletal muscle of a severe hemophilia B patient 10 years after AAV-mediated gene transfer. Blood.

[26] Xu L (2016). CRISPR-mediated genome editing restores dystrophin expression and function in mdx mice. Mol. Ther..

[27] Miller JB (2017). Non-Viral CRISPR/Cas gene editing in vitro and in vivo enabled by synthetic nanoparticle co-delivery of Cas9 mRNA and sgRNA. Angew. Chemie Int. Ed..

[28] Neri M (2007). Dystrophin levels as low as 30% are sufficient to avoid muscular dystrophy in the human. Neuromuscul. Disord..

[29] Dumont NA, Bentzinger CF, Sincennes M-C, Rudnicki MA (2015). Satellite cells and skeletal muscle regeneration. Compr. Physiol..

[30] Arnett AL (2014). Adeno-associated viral vectors do not efficiently target muscle satellite cells. Mol. Ther..

[31] Ronzitti G, Gross DA, Mingozzi F (2020). Human immune responses to adeno-associated virus (AAV) vectors. Front. Immunol..

[32] Charlesworth CT (2019). Identification of pre-existing adaptive immunity to Cas9 proteins in humans. Nat. Med..

[33] Lee K (2017). Nanoparticle delivery of Cas9 ribonucleoprotein and donor DNA in vivo induces homology-directed DNA repair. Nat. Biomed. Eng..

[34] Liu C, Zhang L, Liu H, Cheng K (2017). Delivery strategies of the CRISPR-Cas9 gene-editing system for therapeutic applications. J. Control. Release.

[35] Emami M (2019). Polyrotaxane nanocarriers can deliver CRISPR/Cas9 plasmid to dystrophic muscle cells to successfully edit the DMD Gene. Adv. Ther..

[36] Gee P (2020). Extracellular nanovesicles for packaging of CRISPR-Cas9 protein and sgRNA to induce therapeutic exon skipping. Nat. Commun..

[37] Birnkrant DJ (2018). Diagnosis and management of Duchenne muscular dystrophy, part 1: Diagnosis, and neuromuscular, rehabilitation, endocrine, and gastrointestinal and nutritional management. Lancet Neurol..

[38] Birnkrant DJ (2018). Diagnosis and management of Duchenne muscular dystrophy, part 2: Respiratory, cardiac, bone health, and orthopaedic management. Lancet Neurol..

[39] Mout R (2017). Direct cytosolic delivery of CRISPR/Cas9-ribonucleoprotein for efficient gene editing. ACS Nano.

[40] Lyu P, Javidi-Parsijani P, Atala A, Lu B (2019). Delivering Cas9/sgRNA ribonucleoprotein (RNP) by lentiviral capsid-based bionanoparticles for efficient ‘hit-and-run’ genome editing. Nucleic Acids Res..

[41] Saga S, Hamaguchi M, Hoshino M, Kojima K (1985). Expression of meta-vinculin associated with differentiation of chicken embryonal muscle cells. Exp. Cell Res..

