# Supplementary material for: Transiently expressed CRISPR/Cas9 induces wild-type dystrophin in vitro in DMD patient myoblasts carrying duplications
Source: Sci Rep. 2022 Mar 8;12:3756. doi: 10.1038/s41598-022-07671-w (PMC8904532; doi:10.1038/s41598-022-07671-w)
Supplement: Supplementary file 1 — Supplementary Information. [file 41598_2022_7671_MOESM1_ESM.pdf]

a)

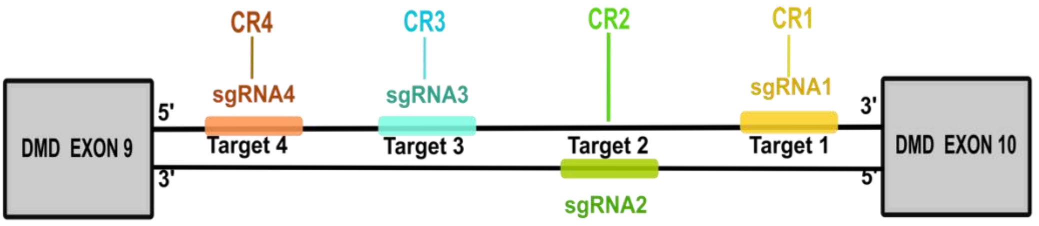

b)

Nuclease	Full lenght amplicon (bp)	Expected cleaved fragments (bp)
CRISPR1 (CR1)	710	437 & 273
CRISPR2 (CR2)	450	281 & 149
CRISPR3 (CR3)	630	373 & 257
CRISPR4 (CR4)	565	371 & 194

c)

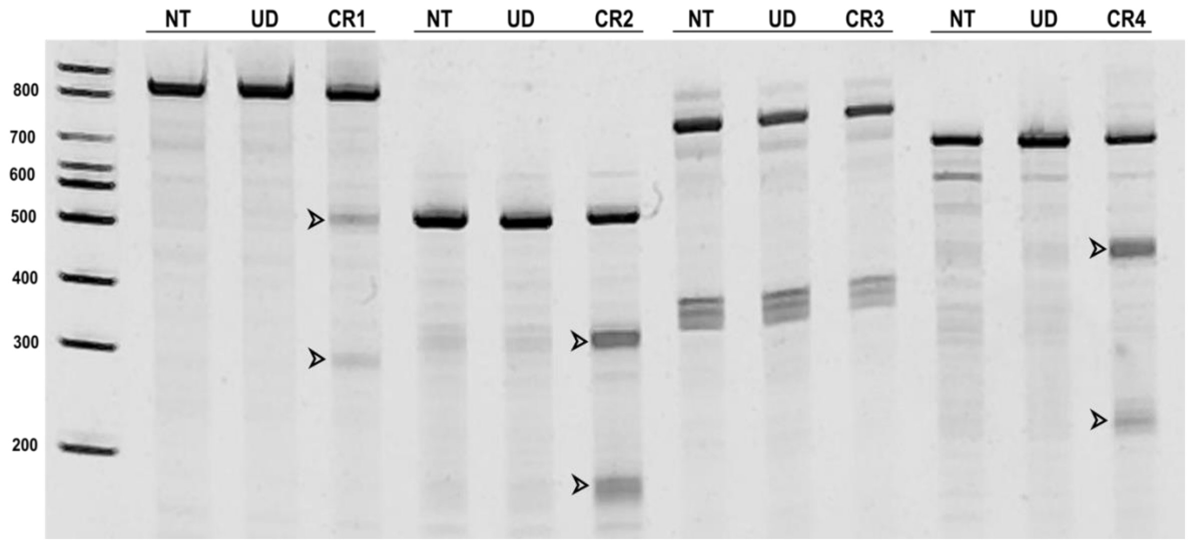

__Supplementary Figure 1: sgRNAs testing in HEK293T cells and identification of the best CRISPR/Cas9 nuclease in HEK293T cells.__

a)sgRNAs designed to target regions within *DMD* intron 9 and cloned in the LentiCRISPRv1 vector to generate functional CRISPR/Cas9 nucleases (CRs). All CRs (named from 1 to 4) recognise sequences in the forward genomic strand, apart for CR2. b) Size of expected full-length and cleaved bands of each CR upon T7E1 assay. c) T7E1 assay representing genomic amplicons obtained upon each of three CRs transfections done in HEK293T cells. Arrows show cleaved bands of the expected size identified in CR1, CR2, and CR4. NT = untreated cells, UD = cells transfected with the undigested LentiCRISPRv1 vector.

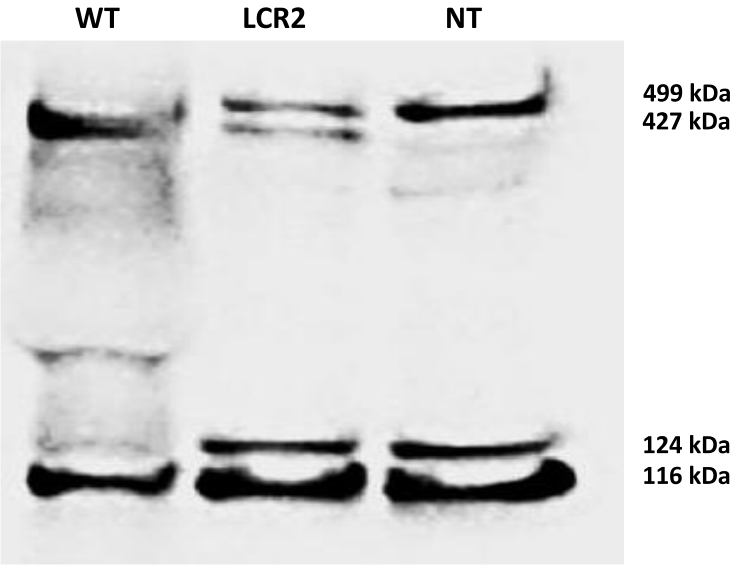

Supplementary Figure 2: Full size image of gel shown in Figure 1i.

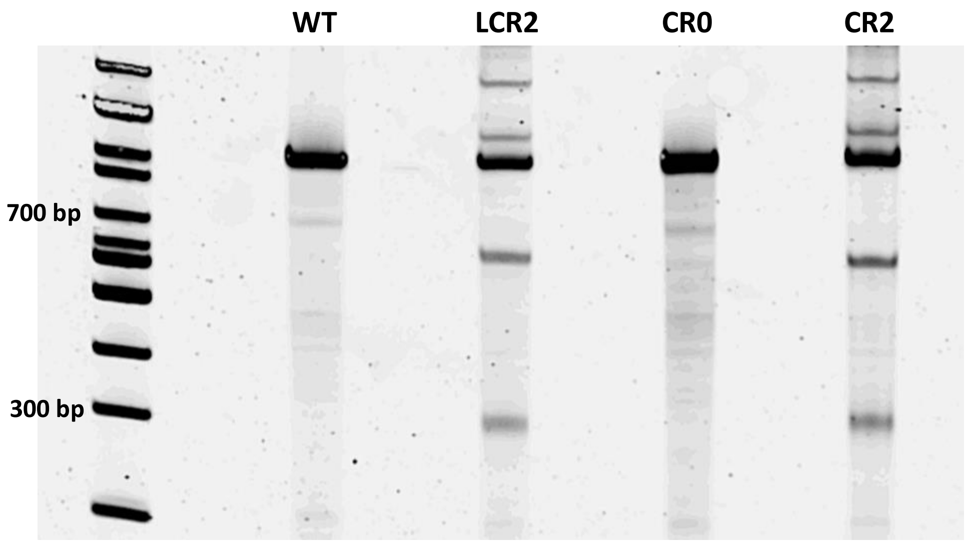

Supplementary Figure 3: Full size image of gel shown in Figure 3b.

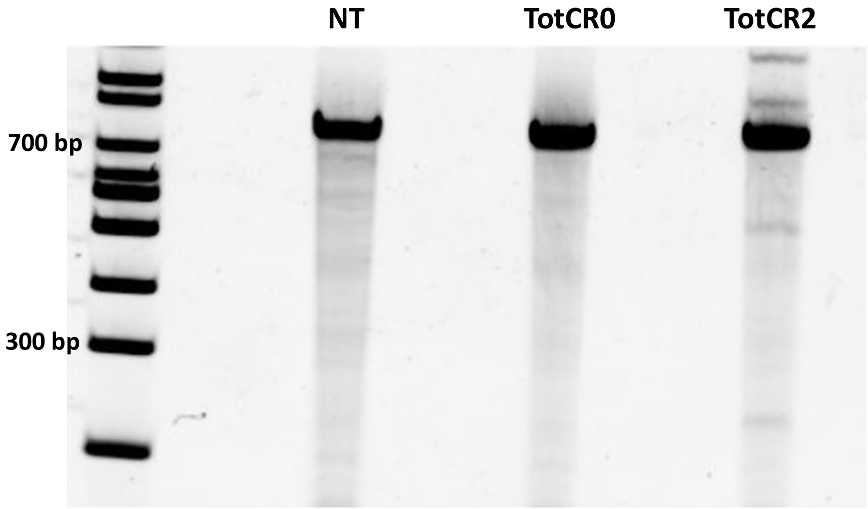

Supplementary Figure 4: Full size image of gel shown in Figure 3d.

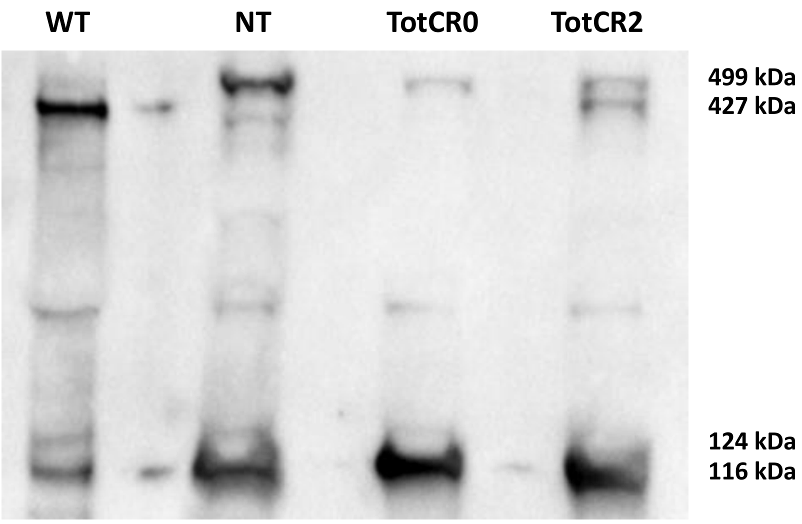

Supplementary Figure 5: Full size image of Western Blot gel shown in Figure 3g.

Supplementary Table 1: *Design of CRISPR/Cas9 nucleases targeting DMD intron 9*. a) DMD intron 9 regions chosen as input for the sgRNAs design algorithm. b) Sequences of sgRNAs cloned in the LentiCRISPRv1 plasmid and their recognized protospacer adjacent motifs (PAMs).

**a) Location and sequence of genomic inputs chosen for sgRNAs design:**	
Intron 9 – sequence 1	32,646,235.CAGCAGACATGTAGTTTATCCCAAGAAGCCTCTCCTGTCACTTG AGTGTATGGTTCCTATAGCATGCAAATCTCTGCAAACATATGGCCAGGTTTAC AGCTCATGTAAACTACCCCGTCTCTATTACTTGGCTTCAGATGGGGAGAAGTT AGAGCCACGCAGAGCGCTCCAGCCTCTCCAGAGGAAGCCTTTCTCACCAGAT TTTTCTAATC GA CCTCCTTGCTT.32,646,010
Intron 9 – sequence 2	32,662,890.AAAATTGCCACAGTATACATTAAATATCTTTGTTTATCCCATTTT AGTTATTTTGTGTGATATCTCTAAATGCTGTTCTGTATATGCTGCTGACAAGG AGCAAATTTATTTGTTTAGAAATTCTGCAGCCTTTCCATCCTTTGCCTTAACTT TCTTGTTGAG GTACTAATGAGCTGTGA.31,656,710
Intron 9 – sequence 3	32,691,476.ATTTCCCTGATAATTCATAATGTTGAACATATTTTCATACACCTG TTGACCATTTGGGTGTCGTCTTTGGAGAAATGTCTGTTCAAGCCTTTAGCCCA TTTTTAATTGCGTTTTTAGTTTTTTTTTTTTTGCCAATGAGTTGTAGGAATTCTT TATATATTTT AGAAGATAACCAG.32,691,300
Intron 9 – sequence 4	32,696,670.TGAGCTGCCTTTTCTAGGTTTTCTCCAATGCCTATTCTGGTAGCA CATTTTCACCTCATCACCACCTTCATTAAGGCATCGCTCAGGTATGACTCCTTA ATGAAGTAATTACAAATGGACTTACATCTTGCTAGGCTCTGCTCATTCTACCT TTGTGTAA ATATTACCAAATACATAAT.32,696,490
**b) sgRNAs chosen to be expressed by CRISPR/Cas9 nucleases**	
sgRNA1	ACTACCCCGTCTCTATTACT (PAM = TGG)
sgRNA2	GTACCTCAACAAGAAAGTTA (PAM = AGG)
sgRNA3	GACCATTTGGGTGTCGTCTT (PAM = TGG)
sgRNA	CCTTCATTAAGGCATCGCTC (PAM = AGG

Supplementary Table 2: List of primers designed to amplify CRISPR/Cas9 target sites in HEK293T cells, DUPmyo and DUPmyo-i myoblasts.

Primer name:	Primer sequence:	Expected amplicon size:
CR1 forward (used in HEK293T)	5’-GACCTCAGCAAAGTCCCTCT-3’	710 bp
CR1 reverse (used in HEK293T)	5’-ACCTCACCCACCCATCTAAA-3’	
CR2 forward (used in HEK293T)	5’-TCAATGGCAGGTGATATCGC-3’	450 bp
CR2 reverse (used in HEK293T)	5’-GGTTGTTCCGTATAGTTGGCC-3’	
CR3 forward (used in HEK293T)	5’-TGCTAGATCAAGTGGGAGTTCT-3’	630 bp
CR3 reverse (used in HEK293T)	5’ACTCAGAGCTATTAGGAGGGT-3’	
CR4 forward (used in HEK293T)	5’TCCCTCCGTTGAAATCACCA-3’	565 bp
CR4 reverse (used in HEK293T)	5’-CTGTAGGGGCAGTGAAAGCT-3	
CR2-NEW forward (used in both HEK293T and myoblasts)	5’ TGGATGCCAAAACCTACAGT 3’	728 bp
CR2-NEW reverse (used in both HEK293T and myoblasts)	5’ GTACGGCAGCTCTTCAAAAA 3’	
